# TB case detection in Tajikistan - analysis of existing obstacles

**DOI:** 10.5195/cajgh.2013.48

**Published:** 2013-10-03

**Authors:** Alexei Korobitsyn, Oktam Bobokhojaev, Thomas Mohr, Jamila Ismoilova, Mavluda Makhmudova, Alex Trusov

**Affiliations:** 1 Project HOPE; 2 National TB Centre, Dushanbe, Tajikistan

**Keywords:** tuberculosis, detection, health services, access

## Abstract

**Background:**

Tajikistan National TB Control Program

**Objective:**

(1) To identify the main obstacles to increasing TB Detection in Tajikistan. (2) To identify interventions that improve TB detection.

**Methods:**

Review of the available original research data, health normative base, health systems performance and national economic data, following WHO framework for detection of TB cases, which is based on three scenarios of why incident cases of TB may not be notified.

**Results:**

Data analysis revealed that some aspects of TB case detection are more problematic than others and that there are gaps in the knowledge of specific obstacles to TB case detection. The phenomenon of “initial default” in Tajikistan has been documented; however, it needs to be studied further. The laboratory services detect infectious TB cases effectively; however, referrals of appropriate suspects for TB diagnosis may lag behind. The knowledge about TB in the general population has improved. Yet, the problem of TB related stigma persists, thus being an obstacle for effective TB detection. High economic cost of health services driven by under-the-table payments was identified as another barrier for access to health services.

**Conclusion:**

Health system strengthening should become a primary intervention to improve case detection in Tajikistan. More research on reasons contributing to the failure to register TB cases, as well as factors underlying stigma is needed.

Tajikistan is a landlocked country in Central Asia, bordering China, Afghanistan, Uzbekistan and Kyrgyzstan, formerly part of the Soviet Union, with a territory of 143.1 thousands sq. km and population size of 6,952,223.^1^ The majority of its territory (93%) is mountainous. According to World Bank data, Tajikistan is the poorest country in former Soviet Union with a Gross National Income (GNI) per capita USD 800.^2^

The country gained independence in 1991. In 1992–1997, however, Tajikistan plunged into civil war. As a result of this fratricidal conflict, various sources estimate that between 40,000 to 100,000 people died, thousands were handicapped, about a million became refugees and internally displaced, more than 50,000 households destroyed, and a damaged economy estimating at a US$7 billion.^3^ The effect of the conflict on health care has not been studied; however, it is logical to assume that the health system infrastructure and human resources suffered along with other parts of a civil society.

Tuberculosis (TB) is among the most important public health problems in Tajikistan. In 2010, the TB notification rate amounted to 92/100,000 for all cases.^3^ World Health Organization (WHO) estimated a TB mortality rate of 41/100,000 in 2010, which ranks Tajikistan highest in the WHO EURO region.^3^

The WHO promoted Directly Observed Therapy Short (DOTS) course strategy implementation was started in 2 pilot districts in 2002 by Project HOPE, international Non-Governmental Organization (NGO), and the support of United States Agency for International Development (USAID). The massive scale-up of the new TB control strategy started in 2004, accelerated with support from the Global Fund to Fight AIDS, Tuberculosis and Malaria, and by the end of 2007, the strategy was implemented in the whole country.

The National TB control strategy adopted in 2010 in Tajikistan is aligned with the Global “STOP TB” strategy and has among its objectives detection of 70% of all existing TB cases in the country and the successful treatment of 85% of cases.^1^

TB detection is primarily taking place in Primary Health Care (PHC) facilities where individuals are evaluated by physicians and undergo sputum smear microscopy if TB diagnosis is suspected. TB reference diagnosis is made on a regional level in tertiary health facilities.

The treatment success rate (TSR) in Tajikistan reached WHO target level (85%) in 2006.^3^ That, however, was attributed to the 40 pilot districts implementing the DOTS strategy (approximately 2/3 of the country population). In 2007, rapid expansion of the DOTS strategy was undertaken, and 100% coverage was achieved within 1 year. Thereafter, there was an observed gradual negative trend in TSR (83% in 2007–2008, 81% in 2009).^3^

There were efforts undertaken to verify quality of the data on treatment outcomes in 2009–2010. Randomly selected primary documentation was verified, with a focus on whether it was complete and sufficient to support the fact of the sputum conversion. In approximately 90% of the cases, the existing primary documentation was judged sufficient to support the quality of the data.^5^

The WHO estimated case detection rate (CDR) in Tajikistan for 2009–2010 was 44% (36–54%) for all cases, which was the lowest in the WHO EURO region.^3^ The trend of low case detection has continued for the last 14 years. From 1995 to 2005 (after civil war ended), there was minimal positive dynamics in case detection rate, 1–2% per year. Even though in 2005 to 2008 there was a period of rapid DOTS strategy scale up, there was no substantial increase in CDR in 2009 (all countries covered by DOTS) or 2010. According to the WHO global TB report in 2010, Tajikistan case detection was substantially lower than average case detection of the central Asian countries (67.2%, *p* < 0.0001), former Soviet countries (75.0%, *p* < 0.0001), and European WHO region in general (87.2%, *p* < 0.0001), thus demonstrating one of the lowest CDR in the world.^3^

## Objective

This article analyzes the underlying reasons for the low TB case detection in Tajikistan. It attempts to answer the following questions: What are the main obstacles for improving TB case detection in Tajikistan? Are those obstacles only within health sector? What interventions can increase case detection?

According to the WHO framework for assessment of TB cases detection, there are three main reasons why incident cases of TB may not be notified: (A) Cases are diagnosed but not reported. For patients in this category, strengthening surveillance systems, establishing links with the full range of heathcare providers for effective information exchange, and corrective measures for early patient default will help. Stronger enforcement of legislation regarding notification of cases (where this is mandated by law) is important as well. (B) Cases seek care but are not diagnosed. For patients in this category, better diagnostic capacity is needed. This means better laboratory capacity as well as knowledgeable and experienced staff, both laboratory technicians and clinicians, especially in peripheral-level healthcare facilities. Normally, this is achieved through quality training, on-the-job mentoring linked to a monitoring system. (C) Cases do not seek care. For people in this category, reasons include not recognizing any symptoms of TB and/or no access (financial or geographic) to healthcare services, or seeking care outside of “official” health services boundaries (i.e. either from traditional healers or privately from known health professionals). The latter option may occur either due to above-mentioned financial/geographical constraints or due to stigma. To reach cases in this category, health systems need to be strengthened so that basic healthcare services are available to more people, and financial barriers to diagnosis (and subsequently, treatment) need to be mitigated or removed. The general population needs to be aware of TB symptoms, prevention, and care principles following evidence-based approach.^6^

In order to understand reasons underlying each of these scenarios, available data from related studies conducted in Tajikistan were analyzed. The analysis was supported by the data from public domains (government strategies and reports, international development data sources).

## Analysis of the Problem

### (A) Cases are diagnosed but not reported

The first possible group of reasons “cases are diagnosed but not reported” may include two options – TB cases may be treated without reporting or not reported due to early default. There is anecdotal data in regard to the former option in Tajikistan; however, no systematic studies were undertaken. The current analysis will focus on the latter option - “initial default.” Substantial attention was given to this phenomenon recently worldwide. In 2008, the “International Journal of Tuberculosis and Lung Diseases” published three original articles on the initial default.^7^–^9^ An initial defaulter is a patient who was detected in a smear microscopy laboratory and consequently recorded in laboratory register as a sputum smear-positive, but was not registered in TB patient register and, hence, did not start treatment.^10^ It is recognized that phenomenon of initial defaulters limits both detection (registration) and treatment outcomes of TB cases in society.^10^ The referenced original studies documented frequency of initial defaulters from 5% (India) to 26% (South Africa).^8^,^9^

The problem of initial defaulters as a contributing factor to the low case detection rate was studied in Tajikistan to a limited extent. As part of routine monitoring visits, the registration of new pulmonary SS+ TB cases was cross-checked in microscopy laboratory register (TB 04) and TB district register (TB 03) in selected districts in Quarters 3 and 4 in 2008 and Quarters 1 and 2 in 2009. During that period, 45 out of 254 (18%) sputum smear positive cases were revealed as unregistered. Of those 45.6% (27) were inhabitants of other districts. The reasons the remaining 18 unregistered cases did not start treatment were: 2 deaths, 1 refused to start treatment, 1 moved out of the country, and 1 was imprisoned. 13 cases (29%) did not start treatment for unidentified reasons.^11^

The analysis of initial defaulters in Tajikistan revealed two important findings: firstly, that people are commonly referred to a different district than the one they reside in for a sputum microscopy test (10.6% of detected SS+ cases were residents of neighboring districts). Secondly, among newly detected SS+ cases who are residents of same districts, initial defaulters have comprised on average 7.9% (18/227), being as high as 25% (13/52) in the least effective DOTS center. The major limitation of this study is that reasons for initial defaults for the large portion of patients were not investigated further.^11^

### (B) Cases seek care but are not diagnosed

This scenario includes TB cases visiting health services but not being diagnosed. There are two principal reasons for that – laboratory service fails to detect *Mycobacterium tuberculosis* or primary health care service fails to refer appropriate suspects for TB laboratory test. We will analyze each of the above-mentioned contributing factors in Tajikistan.

To successfully detect TB cases, better diagnostic capacity is needed. The latter assumes availability of equipment, disposable supplies, qualified laboratory and clinical staff, and effective referral patterns.^3^

Though recent advancements in TB laboratory technologies are promising, sputum smear microscopy is still the primary basis for TB diagnosis, being effective and efficient in detecting most epidemiologically dangerous TB cases (i.e. excreting large amounts of *Mycobacterium tuberculosis*). Even though more sensitive methods are important for increasing TB case finding, smear microscopy remains the cornerstone of TB detection, especially in less developed countries. For these reasons, the article will focus only on sputum smear microscopy.

In March 2010, officially there were 97 designated microscopy laboratories in Tajikistan, roughly 1 per 70,000/population. However in practice, only 92 microscopy laboratories were functioning. The laboratories are generally centralized at the district *(nokhia)* level; however, certain districts have more than one laboratory due to larger population (>100,000) and/or accessibility reasons, (remoteness and/or mountainous terrain). Each TB suspect upon presenting at a microscopy laboratory is recorded in TB laboratory register (TB 04), where test results are recorded.

Diagnosis of TB cases by means of smear microscopy is a priority both for the NTP and international partners in TB control, attracting major financial resources and being a target for a technical support. Between 2005 and 2010, smear microscopy increased both in number and in quality, suggesting these concerted efforts are successful (Table 1).

Effective referral mechanism is another factor contributing to quality TB diagnosis, which consists of a well-performing health workforce and sound management practices.

A well-performing health workforce is one that works in ways that are responsive, fair, and efficient to achieve the best health outcomes possible, given available resources and circumstances (i.e. there are sufficient, competent, fairly distributed staff; they are responsive and productive).^12^

Improving the performance of the health workforce was one of the objectives for Knowledge, Attitude and Practice (KAP) surveys implemented in 2005 and 2008 jointly by the international NGO Project HOPE, WHO office in Tajikistan, Sino Project/Swiss Center for International Health with funds from Global Fund to fight AIDS, Tuberculosis and Malaria (GF) and USAID. In 2008, 185 doctors and 357 nurses working in primary health care have participated in the survey. The health providers were questioned if they have passed formal training in principles of the DOTS strategy. There was an attempt to evaluate their competence as well. Only 43% of doctors and 32% of nurses had attended DOTS courses during the last five years preceding the survey. Regarding the quality of knowledge, 72.4% of physicians, compared to only 29% of nurses, working in primary health care correctly noted that the first step in TB diagnosis is a referral for microscopy examination (Table 2).^13^

Quality of sputum is a critical factor influencing TB diagnosis. The same survey showed nurses have insufficient knowledge about contributing factors to collecting quality sputum samples (Table 3).^13^

Another critical factor for making correct TB diagnosis is following the TB diagnostic algorithm. When TB is suspected (prolonged cough, abnormalities on a chest X-ray) but the smear microscopy result is negative, the diagnostic algorithm indicates prescribing broad-spectrum antibiotic therapy. According to the study results, PHC physicians followed this algorithm only in 38.9% of cases.^13^

### (C) Cases do not seek care

The third group of reasons for not detecting TB cases can be attributed to the situation when “cases are not seeking care.” The following reasons in this group, which are of utmost importance for Tajikistan: (1) stigma, (2) high economic cost of the medical services, and (3) lack or insufficient knowledge of TB symptoms by the general population.

The stigma attached to tuberculosis in many societies has been recognized as a major global cause of the limitations of the World Health Organization’s DOTS strategy for TB control.^14^ In the 1960s, stigma was defined by Goffman as “an attribute that is deeply discrediting” and the stigmatized as “individuals who are negatively regarded by the broader society and are devalued, shunned or otherwise lessened in their life chances.”^15^ Jones et al. proposed that people are stigmatized “when they are found to possess a mark that makes them deviate from a prototype or norm.”^16^

It was broadly studied and proved that, for TB patients, stigma has a major impact on access to health care. It affects healthcare seeking behavior, as people are hesitant or choose not to disclose their symptoms to family members, friends or neighbors for fear of possible isolation and hostility towards them.^15^–^17^

In Tajikistan, stigma related to TB was studied in depth within KAP surveys as well. In 2005, TB patients (n=350) were asked if they noticed a change in attitude among family members toward them once the diagnosis of tuberculosis was revealed. About 15% of the patient respondents noticed deterioration in attitude toward them.^18^ The results of survey in 2008, as well as in 2005, showed that the majority of the respondents believe that TB patients should be isolated (2005–84%, 2008–94.4%). More than 52% of them noted that TB patients should be isolated for the entire period of treatment; 32% - until their health improved and 15% - for contagious period only.^13^,^18^

The manifestation of stigma is more distinct on a personal level. Even though the majority of respondents from the general population in 2008 believed that TB can be cured (84%), at the same time 45.3% of them indicated that they would not accept a former TB patient into their family.^13^ Reasons for refusal to accept people with TB into families were explained by respondents as following: fear to be infected with TB (48.9%), TB can be inherited (25.2%), and risk of re-infection (24.1%). These data correlate with self-reports from TB patients, when over 50% of respondents among TB patients of marital age, both men and women reported difficulties when creating a family and high level of TB related stigma among migrant workers.^13^,^19^

Illness often results in an economic burden for individuals as well as households, especially for socially disadvantaged and impoverished groups of the population.^20^ The negative economic effect is further exacerbated in a situation when social support is missing.^21^ In case of underfunded health services, the private out-of-pocket payments can become an important source of health system financing.^22^,^23^ There is overwhelming anecdotal data of prevalent private out-of-pocket payments for health services in Tajikistan. These facts were confirmed at least by several systematic studies.^13^,^18^,^24^–^26^

The “KAP” surveys conducted in 2005 and in 2008 documented the problem of unofficial payments for health services in Tajikistan along with their possible detrimental effect for TB care.^13^,^18^ In 2008, 1/3 of respondents representing general population stated that TB treatment is provided on a pay basis.^13^ Over 68% stated that key reason of delayed care seeking is shortage or lack of money (in 2005, 67.5%) whereas only 7.3% of them mentioned lack of knowledge of TB symptoms as reason for delayed care-seeking (2005–26%).^13^,^18^

The study on household costs of illness during different phases of TB treatment found that an illness episode cost averaged as high as $1,053 USD, with a peak in early stages of treatment ($145 per month before the start of treatment, $153 per month in an intensive, and $95 in a continuation phase, *p* < 0.0005).^24^ These costs not only seriously compromise affordability, but also have a devastating effect on an household budgets in Tajikistan.^24^

Finally, knowledge of TB symptoms by general population plays an important role whether patients are seeking care, should symptoms of TB occur. It has been proven in a number of countries that educational campaigns among population may increase both use of health services by general population and number of newly detected TB cases.^27^

The comparison of 2008 and 2005 KAP survey results indicate improvement in awareness of TB transmission among the population in the pilot districts. Comparative analysis revealed that 63.7% of respondents in the 2005 survey mentioned cough as a basic TB symptom, versus 84.9% of respondents in 2008. There is also increase in respondents’ knowledge of other TB symptoms. In 2005, over a quarter of respondents could not define any of TB symptoms versus just 7% in 2008. According to 2008 survey results, the majority of respondents (61%) believe that the transmission through air (2005, 25.3%) and commonly used items (27.3%) are the main ways for TB transmission. Taking into account that the latter (contact) mode is not epidemiologically significant, this rate (27.3%) indicates that further public education is needed. While 20% of respondents in 2005 were not aware of the means of TB transmission, in 2008 survey this decreased to 5.2%.^13^, ^18^

## Discussion

Detection of TB cases is an essential component of TB control. Its objective is to identify infectious TB sources in society and treat them, thus discontinuing chain of transmission of TB infection.^28^ While it is considered critical to prioritize efforts to treat earlier detected cases of tuberculosis,^29^ it is also important to look into detecting the highest possible proportion of them in order to achieve sustainable control of the tuberculosis epidemic.

Tajikistan has achieved tangible progress in treatment of TB cases, despite having the lowest TB detection rates in the WHO EURO region (44% in 2010). Therefore, TB detection needs to be strengthened, and new approaches applied in order to further improve the trend of TB case detection.

The analysis reveals that some aspects of TB case detection are more problematic than others, whereas certain gaps in knowledge of specific obstacles to TB case detection exist. Thus, the scenario “cases are diagnosed but not reported” is studied considerably less than others. The phenomenon of treatment without registration and reporting due to various reasons (stigma, deprived access to health services due to either distance or financial affordability, belief in traditional healers) was not studied. Alternatively, there have been attempts undertaken to study and prove the phenomenon of “initial default” in Tajikistan. The preliminary analysis has shown that majority (60%) of initial defaulters are residents of the districts different from those where they seek health care. It is not clear at this stage why this has happened, which may be due to stigma or family connections and needs to be studied. At the same time the system of information exchange between districts requires urgent improvement.

For “cases that seek care but are not diagnosed,” it appeared that laboratory services detect infectious TB cases rather effectively, whereas referral of appropriate suspects for sputum smear microscopy possibly lags behind. The problem may be related to the qualification and performance of the health staff, which is possibly being impacted by training and monitoring or by the effectiveness of referral pattern of the existing TB suspects, integration and collaboration between TB and PHC services.

The scenario “cases do not seek care,” appeared to be explored extensively, with two KAP surveys conducted in 2005 and 2008 as well as several other studies yielding mutually supportive results. Starting from 2002, concerted efforts have been applied for public education in regard to general information on TB, which produced measurable results. Yet, the problem of TB attached stigma persists, being one of obstacles for the effective TB detection in Tajikistan.

High economic cost of health services driven by under-the-table payments was identified as another barrier for access to health care. While a simplistic way of regarding this phenomenon would be to call it “corruption” leading to punitive measures only, deeper assessment, supported by a number of studies, is that viewing informal payments as a characteristic of health system frailty which necessitate health system strengthening rather than police measures. If average official salary of doctors in 2011 was USD $71 (Tajik somony 337),^30^ when health services are severely under-funded, under-the-table payments become a means of survival and are difficult to mitigate through punitive action.

## Conclusion

Low case detection in Tajikistan is a complex problem with barriers to its improvement lying on different stages of the TB diagnostic path within health system domain as well as beyond.

Based on results of this study, it may be plausible to suggest health system strengthening (HSS) activities, broadly defined elsewhere as a logical primary approach to increase of TB detection in Tajikistan.^31^ The information collected and analyzed in this article suggests that suboptimal qualification of health cadres, ineffective management and supervision practices, and suboptimal health funding mechanisms are contributing to losing detected cases as “initial defaulters,” ineffectively referring of TB suspects, and, finally, prohibiting access to health services through high informal payments.

More research on unofficial treatment for TB patients outside of TB services, reasons contributing to “initial default,” especially from the perspective of a patient, and factors underlying TB-related stigma is needed.

## Figures and Tables

**Table 1:** Smear microscopy results in Tajikistan 2008–2011.

	DM (PHC+TB services)	# positive	PR (%)	DM (PHC)	# positive	PR (%)
2008	28619	2900	10	12077	538	4
2009	37175	3568	10	16708	712	4
2010	40712	3896	10	19555	925	5
2011 (9 months)	31705	2774	9	14648	1036	7

*Note:* DM – diagnostic microscopy; PHC – primary health care services; PR – positivity rate.

**Table 2:** Suggested actions of PHC providers toward TB suspects.

	PHC Doctors	PHC Nurses
Referral to sputum collection spot	72.4%	28.9%
Referral to DOTS center	11.4%	32.5%
Referral to TB center	3.2%	30.0%
Referral to X-ray room	13.0%	3.4%
Do not know	0.0%	0.8%
Other	0.0%	4.5%

*Note:* PHC-primary health care services.

**Table 3:** Knowledge of factors influencing the reliability of sputum smear microscopy results.

	PHC nurses, providing correct answer
Controlled sputum collection	40.9%
Following sputum collection techniques	65.3%
Number of sputum samples examined	5.3%
Adequate instructions for patients	14.0%
Qualifications of laboratory technician	17.6%
Other	8.8%

*Note:* PHC-primary health care services.
